# Newborn Screening for CAH—Challenges and Opportunities

**DOI:** 10.3390/ijns7010011

**Published:** 2021-02-13

**Authors:** Natasha L. Heather, Anna Nordenstrom

**Affiliations:** 1 National Newborn Metabolic Screening Programme, Specialist Chemical Pathology, LabPlus, Auckland City Hospital, Auckland 1023, New Zealand; 2 Clinical Research Unit, Liggins Institute, University of Auckland, Auckland 1010, New Zealand; 3 Centre for Inherited Metabolic Diseases, Karolinska University Hospital, SE-171 76 Stockholm, Sweden; anna.nordenstrom@ki.se; 4 Department of Women’s and Children’s Health, Karolinska Institutet, SE-171 76 Stockholm, Sweden; 5 Pediatric Endocrinology Unit, Astrid Lindgren´s Children’s Hospital, Karolinska University Hospital, SE-171 76 Stockholm, Sweden

Newborn screening for congenital adrenal hyperplasia (CAH) using 17-hydroxyprogesterone (17-OHP) as an indicator of disease was first introduced in the 1970s [1]. Prior to this, the female preponderance among clinically detected patients and the cellular architecture of the adrenal glands had led to difficulty in understanding both the inheritance and pathogenesis of CAH. Following the introduction of screening, it became clear the CAH is a monogenic autosomal recessively inherited disorder with a wide spectrum of phenotypic severity. The female preponderance was explained by neonatal mortality due to early salt crises or hypoglycemia, mainly amongst boys without obvious signs of the disease at birth. Conversely, as affected females undergo prenatal genital virilisation, they are often, but not always, detected clinically soon after birth.

Newborn screening for CAH detects babies affected by deficiency in the adrenal enzyme 21-hydroxylase, which is the most common cause of CAH (>90%). The molecular genetics of *CYP21A2* and the genotype–phenotype correlation of pathogenic variants have been important tools both in studies of the disease and in setting up relevant and effective screening programs. Most screening programs today aim to prevent neonatal salt crisis and mortality. Hence, they are targeted to detect babies with the classic forms of CAH, mainly the salt-losing form. Newborn screening for CAH provides clear benefit in preventing neonatal salt crisis and mortality, especially amongst boys. However, despite substantial benefits and a high uptake of CAH screening worldwide, there are still a number of ongoing issues to be resolved, including:

- Timing of the specimen collection. This is important in CAH, as the result has to be communicated early enough to be effective in preventing salt crisis, yet later samples can detect more affected individuals, albeit with less severe forms of the disease.
- The relatively high false positive rate and a low positive predictive value (PPV) of CAH screening. This is largely due to variation in 17-OHP levels in the neonatal period and in severely ill infants. In particular, immunoassay measurements of 17-OHP amongst preterm infants can be falsely elevated as a result of cross-reactivity with metabolites found in the immature adrenal gland.
- Clinical follow up and the availability of treatment. This is crucial to a successful screening program. Despite hydrocortisone being an inexpensive medication, it is not readily available in all parts of the world.

These issues are not straightforward and continue to be obstacles both to the uptake of CAH new-born screening and to quality improvement in established programs. They require balanced solutions that reflect local healthcare systems and priorities and may, therefore, be resolved in different ways around the world.

On the initiative of the *International Journal of Newborn Screening* (IJNS), the opportunity arose to realise this project, a Special Issue on “CAH Newborn Screening—Challenges and Opportunities”. The aim of this Special Issue was to describe the current state of CAH screening around the world, with a focus on efforts to find solutions to obstacles and on successful strategies to improve the efficiency of screening.

We would like to take this opportunity to thank the authors for their excellent contributions and the *IJNS* for their support. We feel the resulting series of articles provides an evaluation of the current status of new-born screening for CAH, and insight into the path for quality improvement across the globe.

Dabas and colleagues [2] report on the challenges and opportunities for CAH NBS in India. India’s high annual birth rate results in over 4 million new-borns each year. To date, the only Indian state to provide comprehensive new-born screening is Kerala, a state in Southern India, which commenced screening for CH, CAH, G6PD and galactosaemia in 2012. The incidence and prevalence of CAH in India is not known, although screening pilot studies have reported a variably high incidence of 1:2300 to 1:10,000. Limitations revealed by Indian pilot studies of CAH NBS include high false positive rates, incomplete follow-up of babies with positive screens and limited availability of confirmatory testing and follow-up care. In addition, limited access to maternity care and medical review of new-born babies mean that birth weight or gestational age related cut-off levels, which can generally reduce the false positive rate, cannot easily be applied. The authors describe cultural reasons to conceal gender ambiguity and case series, suggesting high mortality and morbidity amongst those presenting with adrenal crises in infancy, such that the early detection and treatment of affected babies would be expected to greatly improve survival.

The national Swedish neonatal screening programme began screening for CAH in 1986 and has made significant contributions to the global understanding of CAH. In a further review, Lajic and colleagues [3] report that long-term outcomes for patients with CAH can be improved by close collaboration between the screening laboratory and the clinical side. This is because good outcomes from new-born screening do not just relate to early detection and are highly dependent on clinical follow up. The authors demonstrate how the screening laboratory can contribute to awareness of CAH amongst clinicians, to knowledge and information about the disease, and to initial treatment of screen-detected babies with CAH. This collaboration is important in ensuring prompt and sufficient early treatment, as well as to avoid overtreatment. Furthermore, the Swedish National CAH Registry contains data on over 700 individuals with CAH and provides valuable information to support screening evaluation through notification of missed cases or delayed diagnoses, as well as through assessment of long-term outcomes. Long-term results regarding mortality, cardiovascular outcome, cognition and possibly fertility may similarly be improved.

Held and colleagues [4] provide a review of the impact of foetal and neonatal physiology on new-born screening for CAH. Current first-tier screening methodologies lack specificity, leading to a large number of false positive cases, and adequate sensitivity to detect all cases of classic 21OHD that would benefit from treatment. First-tier screening, based on immunoassay assessment of blood 17-OHP levels in new-borns lacks specificity, especially amongst preterm babies, leading to a high rate of false positive screen results. Sensitivity may also be inadequate to detect all cases that would benefit from early detection and treatment. The pathology of CAH due to 21-hydroxylase deficiency, the development of the foetal hypothalamic-pituitary-adrenal axis development and adrenal steroidogenesis are all described. The authors highlight five factors that contribute to both false positive and false negative results, reviewed alongside current best practice for specimen collection in the United States and worldwide.

Specific problems that arise for children with CAH in resource poor countries are outlined in the publication by Armstrong et al [5]. Since 2004, Caring and Living as Neighbours (CLAN), an Australian non-governmental organization working to improve the lives for children with chronic health conditions in resource poor countries, has collaborated with a broad range of partners across the Asia–Pacific region in order to improve the quality of life of children with CAH. The treatment of CAH is complex in that steroid doses must be adjusted during infections with a fever and may require hospital care for intravenous treatment in severe situations. Common issues for children in resource-poor countries include access to medication, education regarding medical management, and access to family support groups. This study shows that improvements in quality of life for children with CAH can be achieved using CLAN’s approach.

An update of Swedish neonatal screening [6] illustrates the genotype–phenotype correlation in CAH and how that can be used to improve the screening and understanding of CAH. The detection of virtually all patients with the salt losing form and all of those with the null genotype demonstrate the effectiveness of new-born screening. The Swedish paper also illustrates that samples collected later, especially amongst preschool children, facilitate the detection of less severe forms of the disease. Offering screening to children up to the age of 8 years when moving into Sweden led to the identification of a surprisingly high number of children with non-classic and simple virilising forms. These children are obviously not the target of the new-born screen, and the paper raises a valid question regarding whether such children should then be treated with replacement doses of hydrocortisone.

New-born screening for CAH is now performed in all states in the USA, with specimen collection occurring relatively early, and most often between 24–48 h after birth. Furthermore, the Secretary’s Advisory Committee on Heritable Disorders in Newborns and Children (ACHDNC) recommends a target of notifying presumptive positive screening results for CAH and other time-critical conditions by five days of life [7]. The Special Issue includes an analysis of screening performance, particularly timeliness, across the USA, based on voluntary data submitted to the Newborn Screening Technical assistance and Evaluation Program (NewSTEPS) data repository [8]. Their report includes > 850 aggregate case counts of CAH and nearly 500 with individual case level data, submitted from 35 contributing state screening laboratories. A key strength of the NewSTEPS data is the use of public health surveillance case definitions to facilitate the consistent categorisation of new-borns. Of note, the median age at release of out-of-range CAH screening results was just 4 days, reflecting considerable success in initiatives to extend laboratory operating hours and facilitate the rapid reporting of out-of-range results.

Within the USA, screening protocols have been adapted and implemented separately by individual state laboratories over the past several decades. The Special Issue includes an overview of 17 of the screening protocols currently used in the USA, illustrating the presence of large differences in approach, as well as screening sensitivity and specificity [9]. Despite using birth-weight-related cut-off levels, reported PPV varied from less than 1 to 50%, and CAH prevalence from 1/10,000 to nearly 1/30,000. Although there was considerable variation in 17-OHP cut-off levels, this did not correlate to either PPV or prevalence. Most state screening laboratories recommend a second sample for infants in intensive care. In addition, there are four two-screen programmes which require a second sample to be collected at two weeks of life, regardless of the result of the first sample, and PPVs were highest amongst these. The authors recommend that quality improvement be directed at the standardisation of protocols across state laboratories, as well as towards enhanced communication between clinicians and screening laboratories, with the eventual aim of achieving a uniform set of best practices.

Within this Special Issue, two Australasian screening programmes report their experience in implementing a second-tier liquid chromatography tandem mass spectrometry (LC-MS/MS) method. De Hora and colleagues from New Zealand [10] report on the performance of a LC-MS/MS method to measure 17-OHP alone. Screening performance was assessed by comparing national screening metrics 2 years before and after LC-MS/MS implementation, within a screened population of approximately 60,000 each year. In the 2 years after LC-MS/MS implementation, the authors demonstrated a four-fold reduction in the overall number of false positive screening tests and concomitant increase in PPV from nearly 2 to 11%, with no loss of sensitivity observed. Furthermore, they noted that the LC-MS/MS method could be turned around and reported quickly after an initial elevated primary immunoassay test, with results available within 2 h when required. 

New South Wales was the first Australian state to commence NBS for CAH in 2018, also using a two-tier protocol. The results of the first 203,000 screened babies are reported here [11]. Samples above the 17OHP threshold level on first-tier immunoassay were further tested using LC-MS/MS, and those with a ratio of (17OHP + androstenedione)/cortisol > 2 and/or 17OHP > 200 nmol/L on LC-MS/MS were referred to as presumptive positive screens. The incidence of CAH detected through NBS was 1:22,551, with a PPV of >70% and no known false negatives. All affected newborns were notified and reviewed clinically prior to the development of an adrenal crisis. In contrast to high false positive rates still reported through screening protocols that rely solely on 17OHP immunoassay levels, this report demonstrates that screening specificity can be dramatically improved through the use of a steroid profile.

We want to thank all the authors for the contributions to this Special Issue, and the expert reviewers for their valuable comments and questions, which were important to the overall quality of the series. New-born screening for CAH has been shown to be effective in detecting infants at risk of developing adrenal salt crisis and thereby decreasing neonatal mortality. It is possible that the screening per se also improves the long-term outcome for these patients in several ways, including increasing knowledge of the disease and its treatment. The authors have provided a state-of-the-art series to help achieve these goals across the globe. Whilst much has been achieved in the field of NBS for CAH, there is clearly still work to do in optimising both the screening approach and outcomes.
